# Supplementary material for: Ability of Garlic and Ginger Oil to Reduce Salmonella in Post-Harvest Poultry
Source: Animals (Basel). 2022 Oct 29;12(21):2974. doi: 10.3390/ani12212974 (PMC9656020; doi:10.3390/ani12212974)
Supplement: Supplementary file 1 [file animals-12-02974-s001.zip › animals-1974711-supplementary.pdf]

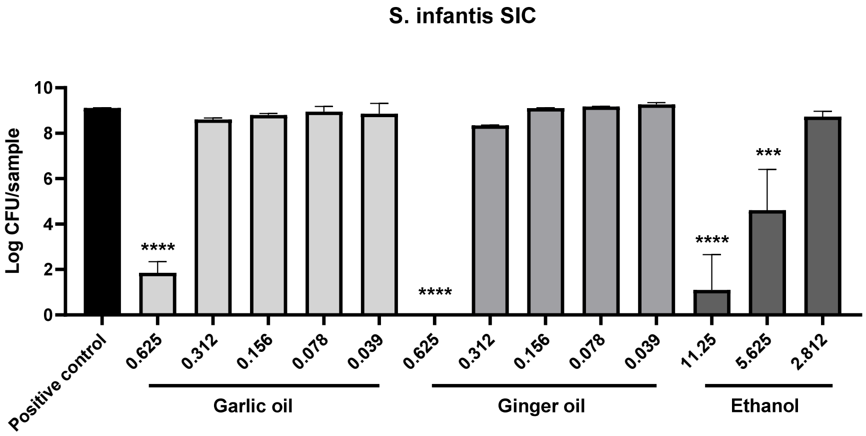

Figure S1: Determination of SIC of garlic oil, ginger oil and ethanol against *S.* Infantis in a microtiter plate. Results are averages of two independent experiments, each contains duplicates samples (mean and STD). ***P<0.001; ****P<0.0001.
